# A Subset of PD-1-Expressing CD56^bright^ NK Cells Identifies Patients with Good Response to Immune Checkpoint Inhibitors in Lung Cancer

**DOI:** 10.3390/cancers15020329

**Published:** 2023-01-04

**Authors:** Marta Gascón-Ruiz, Ariel Ramírez-Labrada, Rodrigo Lastra, Luis Martínez-Lostao, J. Ramón Paño-Pardo, Andrea Sesma, María Zapata-García, Alba Moratiel, Elisa Quílez, Irene Torres-Ramón, Alfonso Yubero, María Pilar Domingo, Patricia Esteban, Eva M. Gálvez, Julián Pardo, Dolores Isla

**Affiliations:** 1 Medical Oncology Department, University Hospital Lozano Blesa, 50009 Zaragoza, Spain; 2 Aragon Health Research Institute (IIS Aragón), 50009 Zaragoza, Spain; 3 Nanotoxicology and Immunotoxicology Unit (IIS Aragón), 50009 Zaragoza, Spain; 4 CIBER de Enfermedades Infecciosas (CIBERINFEC), 28029 Madrid, Spain; 5 Immunology Department, University Hospital Lozano Blesa, 50009 Zaragoza, Spain; 6 Department of Microbiology, Pediatrics, Radiology and Public Health, University of Zaragoza, 50009 Zaragoza, Spain; 7 Aragon Nanoscience Institute, 50018 Zaragoza, Spain; 8 Aragon Materials Science Institute, 50009 Zaragoza, Spain; 9 Infectious Disease Department, University Hospital Lozano Blesa, 50009 Zaragoza, Spain; 10 Instituto de Carboquímica (ICB-CSIC), Miguel Luesma 4, 50018 Zaragoza, Spain; 11 Microbiology, Radiology, Pediatry and Public Health Department Medicine, University of Zaragoza, 50009 Zaragoza, Spain

**Keywords:** T lymphocytes, NK cells, lung cancer, immunotherapy, biomarkers

## Abstract

**Simple Summary:**

In recent years, studies in cancer immunotherapy have focused on finding predictive response biomarkers to anticipate which patients will respond to each kind of treatment, optimizing treatment strategy and reducing toxicities and costs. Unfortunately, these studies are still limited, and no conclusive results have been obtained. This study was designed to prospectively analyze a cohort of lung cancer patients receiving immunotherapy attempting to determine cellular biomarkers of response, as T-lymphocyte and natural killer (NK) cell subsets, in peripheral blood. Despite previous studies, none of these populations have been identified yet with clinical relevance to be established in clinical practice. In our study, we have identified a subset of circulating CD56+CD16-PD-1+ NK cells showing a good predictive ability. These results contribute to the development of precision medicine protocols where we could stratify patients according to the expected treatment response to choose the best option for each patient.

**Abstract:**

(1) Despite the effectiveness of immune checkpoint inhibitors (ICIs) in lung cancer, there is a lack of knowledge about predictive biomarkers. The objective of our study is to analyze different subsets of T-lymphocytes and natural killer (NK) cells as predictive biomarkers in a cohort of patients with nonsmall cell lung cancer (NSCLC) treated with ICI. (2) This is an observational, prospective study with 55 NSCLC patients treated with ICI. A total of 43 T and NK cell subsets are analyzed in peripheral blood, including the main markers of exhaustion, differentiation, memory, activation, and inhibition. (3) Regarding the descriptive data, Granzyme B+CD4+ Treg lymphocytes stand out (median 17.4%), and within the NK populations, most patients presented cytotoxic NK cells (CD56+CD3−CD16+GranzymeB+; median 94.8%), and about half of them have highly differentiated adaptive-like NK cells (CD56+CD3−CD16+CD57+ (mean 59.8%). A statistically significant difference was observed between the expression of PD1 within the CD56^bright^ NK cell subpopulation (CD56+CD3−CD16−PD-1+) (*p* = 0.047) and a better OS. (4) Circulating immune cell subpopulations are promising prognostic biomarkers for ICI. Pending on validation with a larger sample, here we provide an analysis of the major circulating T and NK cell subsets involved in cancer immunity, with promising results despite a small sample size.

## 1. Introduction

In recent years, great advances have been made in the field of cancer immunology, confirming the importance of immune cells in the recognition and control of tumor growth and the application of this knowledge to the development of therapies based on the modulation of the immune response, known as immunotherapy [1].

CD4+ and CD8+ T lymphocytes and NK cells are the most critical cells involved in the control of tumor development [2,3], although they employ different mechanisms to recognize and eliminate cancer cells.

Numerous studies have shown that lymphocytes and NK cells are key to immune defense against tumor cells. Several cell populations have been shown to be involved, such as CD4+T, Treg and NKT [4,5,6,7].

T cells recognize antigens presented with HLA molecules, while NK cells are activated by a highly regulated balance between inhibitory and activator signals recognized by different inhibitory and activating NK cell receptors [8]. Among the different receptors controlling NK cell activation, two main groups are identified according to their functionality: the inhibitory receptors that recognize different HLA (Human Leukocyte Antigen)-I molecules and the activating receptors recognizing ligands expressed by cells during stress, infection or transformation. In contrast, primary T cells’ responses are organized by antigens presented in the context of HLA-II molecules expressed by antigen-presenting cells (mainly dendritic cells) to CD4+ T cells that will regulate the activation of antigen-specific CD8+ T cells after they recognize antigens expressed by HLA-I together with other costimulatory signals provided by dendritic cells. In this way, antigen-specific CD8+ cytotoxic T cells will be generated, and they will recognize and destroy tumor target cells expressing the HLA-I/antigen complex against which they were originally activated. In both cases, NK and T cytotoxic cells will execute tumor cells employing the same cytotoxic mechanisms, mainly the death receptor system (i.e., Fas/FasL) and granule exocytosis [9]. The latter one involves the delivery of granzymes, i.e., GzmB, from effector cell granules to target cell cytosol through target cell membrane pores formed by the pore-forming protein perforin, also present in cytotoxic cell granules. In addition to their ability to kill target cells, GzmB expression in NK/T cells can be used as a marker of cell activation indicating the generation of NK and/or Tc cells with potential antitumor activity. In addition to NK and Tc cells, GzmB expression has been found in other hematological and nonhematological cells, including mast cells, neutrophils or Treg cells [10]. Indeed, it was shown that GzmB was used by Treg cells as an immunosuppression mechanism preventing Tc and NK cell activation against cancer cells [11].

Despite the differences regarding the requirement of specific antigens, in both NK and T cells, the activation process is tightly regulated by a complex network of known activator and inhibitory signals and immune checkpoints (IC). These signals will be integrated by T/NK cell receptors specific for soluble molecules such as cytokines or membrane-associated costimulatory molecules of coinhibitory ligands [12]. These mechanisms are key to regulate the activation of the immune response against foreign material and/or that which poses a threat to the organism, while preserving tissue homeostasis by preventing response to healthy tissues or commensal flora. However, during the process of tumor development, cancer cells have learned to exploit these regulatory mechanisms to their own benefit, acquiring the so-called immune evasion mechanisms, mainly through the expression of molecules that inhibit the antitumor immune responses indicated above [13]. Some of the most studied and better characterized, due to their importance in cancer immunity and immunotherapy, have been CTLA-4 (lymphocyte cytotoxic antigen-4) and PD-1 (programmed cell death-1). Among other functions, binding of CTLA4 and PD-1 to their respective ligands, CD80/86 and PD-L1/2, has a dual protumoral effect. On the one side, it directly inhibits the activation of antitumoral specific T-cell responses, and, on the other hand, it increases the production of regulatory CD4+ T cells (Treg), which in turn promotes immune suppression and decreases the activity of T cytotoxic (Tc) cells and NK cells [10,14,15].

Up to date, the most relevant and successful immunotherapy treatments have been aimed at blocking PD-1/PD-L1 and CTLA-4/CD80-86 interaction, thus enabling reactivation of Tc cell activity against cancer cells. The most used monoclonal antibodies in lung cancer are anti-PD-1, anti-PD-L1 and/or anti-CTLA-4.

Despite the big potential of immunotherapy, there are still difficulties to select those patients who will respond to treatment, as not all patients and not all tumors respond in the same way.

Recently, studies in immunotherapy have focused on finding predictive response biomarkers to anticipate which patients will respond to each type of treatment, avoiding unnecessary toxicities and reducing costs [16]. Many of these studies have explored the possibility that the different types of immune cells present in the tumor microenvironment (TME) and their distribution in peripheral blood may contribute to predicting a good response to immunotherapy for lung cancer.

Unfortunately, the number of studies related to biomarkers of response to immunotherapy is still limited, and no conclusive results have been obtained. Therefore, further work is needed to confirm the potential of circulating immune cell subsets as predictive biomarkers of response to immunotherapy.

Thus, this study was designed to prospectively analyze a cohort of lung cancer patients receiving immunotherapy and to attempt to determine cellular biomarkers in peripheral blood that are predictive of response, focusing on some of the main cells involved in cancer immunity and response to immunotherapy, namely, CD4+ and CD8+T, and NK cell subsets.

## 2. Materials and Methods

### 2.1. Ethical Considerations

This study was evaluated and approved by the Clinical Research Ethics Committee of Aragón (CEICA) with code (C.I. PI19/052). All patients that were included gave their informed consent prior to their inclusion in the study.

### 2.2. Population and Inclusion and Exclusion Criteria

All patients with nonsmall cell lung cancer (NSCLC) who were referred to the medical oncology clinic for immunotherapy treatment between April 2019 and October 2020 were consecutively included.

All patients with unresectable stage III or stage IV NSCLC who were going to start immunotherapy treatment were included. Patients with unresectable stage III will start immunotherapy after receiving concomitant chemotherapy and thoracic radiotherapy without progressive disease. The main exclusion criteria were histology other than NSCLC, concomitant tumor of other origin, autoimmune disorders contraindicating immunotherapy, previous immunotherapy for this tumor or because of any other reason and a grade 2 or higher in the Eastern Cooperative Oncology Group (ECOG) scale. Patients on corticosteroid treatment with doses higher than 10 mg/24 h of prednisone or equivalent were also excluded. Patients with known immunodeficiencies (including HIV infection) were excluded too.

### 2.3. Study Protocol

This is a prospective, longitudinal, noninterventional, single-center study. It was conducted at the Medical Oncology Department of the Lozano Blesa University Hospital, a tertiary hospital in Zaragoza (Spain).

All patients underwent blood sampling at the baseline visit, prior to the start of immunotherapy treatment. At this visit, clinical and demographic variables were also collected by direct interview with the patient.

The degree of functional capacity was defined using the validated ECOG scale [17], with grade 0 being normal capacity and grade 4 being a patient who is bed-ridden all day. Tumor stage was defined according to the grades established by the 8th consensus [18].

Tumor response was established according to the RECIST criteria (v.1.1) [19].

Treatment with immunotherapy was decided at the discretion of the physician responsible for the patient, in accordance with the clinical practice guidelines in force at the time [20,21,22].

Subsequently, clinical follow-up of the patient was carried out in accordance with standard clinical practice protocols (every 2–3 weeks during treatment administration depending on the type of treatment, and every 2–3 months without therapy). This follow-up continued until the patient’s death.

### 2.4. Sample Type and Processing

Samples and data from patients included in this study were provided by the Biobank of the Aragon Health System (integrated in the Spanish National Biobanks Network (PT20/00112)), and they were processed following standard operating procedures with the appropriate approval of the Ethics and Scientific Committees.

#### 2.4.1. Sample Processing

Peripheral blood was collected in heparin sodium tubes and then centrifuged for at least 10 min at room temperature at 2600 revolutions per minute (rpm). In this way, the cell fraction was separated from the plasma, which was extracted with cell pellet and stored at −80 °C for later use.

The cell pellet was diluted in cell medium for human cell culture (RPMI medium 1640) using a 1:1 ratio and added to Histopaque medium-1077 (Sigma, Darmstadt, Germany). After centrifugation at 2500 rpm for 10 min, lymphocyte and other peripheral blood mononuclear cells (PBMC) were isolated. This layer of cells was then washed with RPMI 1640 and divided into aliquots for subsequent staining and analysis by flow cytometry.

#### 2.4.2. Staining, Antibody Panels and Flow Cytometry

For cell surface staining, PMBCs were suspended in 50 µL of phosphate-buffered saline (PBS) with 5% fetal bovine serum (FCS). They were stained with the different antibody panels for 20 min at 4 °C in the dark and washed a second time with PBS + 5% FCS. Finally, they were mixed for 30 min at 4 °C in the dark using 2% paraformaldehyde.

Those cells requiring intracellular staining were permeabilized for 30 min at 4 °C in the dark using a FoxP3 transcription factor buffer kit (Miltenyi). Subsequently, cells were washed twice with permeabilization buffer and suspended in 50 µL of permeabilization buffer with subsequent staining and fixation with intracellular antibody for 30 min at 4 °C in the dark. All samples were processed with Gallios (Beckman Coulter) Flow Cytometer.

The list of antibodies used for immune cell phenotyping is detailed in Table S1.

### 2.5. Determination of Cell Populations in Peripheral Blood

The following populations and ratios in peripheral blood were determined by flow cytometry: (1) CD8+ T-lymphocyte populations (PD-1, TIM3, LAG3, GzmB+) in peripheral blood sample by flow cytometry. (2) Ratio of CD8+ T lymphocytes to CD4+ T lymphocytes in peripheral blood sample by flow cytometry. (3) NK cell populations (CD56, CD3, CD16, KIR, KLRG1, CD94/CD56, CD3, PD-1, LAG3, TIM3, CD57, CD69/CD56, CD3, CD16, NKp44, NKp46, NKG2D, NKG2A, GzmB+, CD57). (4) Effector Memory CD8+ T cells (CD8, CD45RO, CCR7, CD27, CD57). The flow cytometry gating strategy can be seen in Figure S1.

### 2.6. PDL-1 Expression in Lung Biopsies

PD-L1 staining was performed on lung tissue by a pathologist with expertise in lung pathology. Samples were considered suitable for PD-L1 staining if they had more than 100 assessable neoplastic cells. Samples were fixed in 4% formaldehyde, buffered and embedded in paraffin. Later, they were cut into 4 μm thick tissue fractions and stained with haematoxylin-eosin and incubated with anti-PD-L1 (E1L3N^®^) XP^®^ RabbitmAb monoclonal antibody on Ventana’s automated Benchmark ULTRA system. PD-L1 expression was assessed based on a manual count of the percentage of neoplastic cells with partial or complete membrane staining, irrespective of their intensity. PD-L1 is considered negative if it is expressed in a percentage of less than 1%. PD-L1 positive has been divided into expression greater than 50% or between 1 and 50%.

### 2.7. Statistical Analysis

A sample size of 50 patients plus 10% (5 patients) for possible losses was defined, for a total of 55. The sample calculation is based on exploratory criteria for initial signs of scientific evidence in view of the scarcity of available information.

Continuous variables were expressed as median and standard deviation. Qualitative variables were expressed as percentages. The Mann–Whitney U test or Student’s *t*-test were used depending on whether the distribution of the variable was normal or not to test the hypothesis. The normality of the distributions was tested using the Kolmogorov Smirnov test.

Spearman’s *p* test was used to perform the correlation study for samples with a non-normal distribution.

Survival analysis was carried out using Kaplan–Meier curves using the Log-rank test to determine statistical significance in the comparative analyses. The impact of the different covariates on survival was assessed using a Cox regression model.

Probability (*p*) values < 0.05 were considered significant.

## 3. Results

### 3.1. Descriptive Analysis

#### 3.1.1. Patient and Tumor Disease Characteristics

A total of 55 patients (70.9% male) with a median age of 65 years were recruited. Most patients (96.4%) were active smokers or ex-smokers. Most patients (65.5%) had an ECOG of 0. The most frequent tumor stage was stage IV (70.9%), and the most frequent histology was nonsquamous (60%). The rest of the descriptive variables of the cohort are shown in in Table 1.

#### 3.1.2. Determinations in Lymphocyte Populations

The distribution of T lymphocyte populations analyzed is detailed in Table 2. The different T cell subsets were analyzed using the indicated markers in the T cell population and were gated as CD3+CD56− cells. To differentiate between activated and exhausted T cells, the expression of the different checkpoints was analyzed in both GzmB-expressing cells (activated cells) and nonexpressing cells (exhausted cells). In this way we differentiated activated (GzmB+ TIM3+/PD1+/LAG3+) from exhausted cells (GzmB- TIM3+/PD1+/LAG3+). Effector memory (em) T cells were gated as CD45RO+CCR7+CD27−CD57− cells, which were previously found to be expanded intratumorally in metastatic melanoma patients responding to anti-PD1 therapy [23].

As can be seen in Table 2, the distributions of CD8+T and CD4+T lymphocytes were 28.5% and 29.6%, respectively. Analyzing CD8+T lymphocytes, more than 30% expressed GzmB+, indicating a high level of activation. When activated cells were analyzed for expression of inhibitory checkpoints, within the GzmB+ cells, it was found that less than 2% of activated cells expressed either TIM3+, PD1+ or LAG3+. Exhausted cells correspond to 68.3% of TCD8+ cells. Within this population, there was a higher expression of inhibitory checkpoints such as LAG3+ (6.5%) or PD1+ (4.7%). In general, these results suggest that circulating CD8+ T cells in these patients mainly presented an exhausted phenotype, since higher frequencies of IC+ GzmB− cells than IC+ GzmB+ cells were found.

Effector memory CD8+ T lymphocytes accounted for a low percentage, 3.5%. We only focused on this memory subset, as it was previously shown that its presence of TILs correlated with a PD1 response in melanoma patients. In addition, within the T CD4+ subset, a total of 6.5% of Treg cells was found, of which around 17.5% expressed GzmB, suggesting an immunosuppressive potential.

#### 3.1.3. Determinations in NK Cell Populations

Within NK cells, a differentiation was made between NK cell populations with cytotoxic capacities, called CD56^dim^ (CD16+) and NK cells with lower cytotoxic activity but showing regulatory capacities, called CD56^bright^ (CD16−). In addition, markers of activation (GzmB) of highly differentiated cells (CD57+) as well as expression of activating (NKp46+, NKG2D+) and inhibitory receptors (NKG2A+, PD-1+, TIM3+, LAG3+) were analyzed.

The NKT cell population (CD56+CD3+) was been studied, which, despite being a type of T cell, was included in this section, as it also shares characteristics with NK cells and are more related to the innate immune system.

The different NK cell populations analyzed are detailed in Table 3. NK and NKT cells were gated as CD56+CD3− and CD56+CD3+, respectively.

As shown in Table 3, and in line with previous studies including healthy and disease cohorts, most circulating NK cells corresponded to the cytotoxic CD56^dim^ (CD16+) subset (median 93.3%). In addition, all patients showed a high presence of activated NK cells within the CD56^dim^ (CD16+), as shown by the high frequency of GzmB+ cells (median 94.8%). In contrast to the results obtained in T cells, the percentage of activated CD56^dim^ NK cells (GzmB+) expressing different checkpoints (LAG3, TIM3, PD1) was much higher than the corresponding exhausted populations (GzmB−). This result suggests a mobilization of activated NK cells expressing the different ICs with a preponderance of TIM3 and LAG3 versus PD1.

In terms of activator and inhibitory receptor and differentiation marker expression, there was a higher frequency of CD56^dim^ NK cells expressing activating receptors NKG2D (69%) and NKp46 (75%) than those expressing the inhibitory receptor NKG2A (46%). In addition, almost 60% of CD56^dim^ NK cells expressed the highly differentiated marker CD57.

Regarding the NK CD56^bright^ subset (CD16−), a high proportion of the cells did not express GzmB (around 65%), confirming the regulatory nature of these cells. Regarding IC expression profile, and similarly to CD56^dim^ cells, TIM3 was the IC that presented a higher expression.

As is already known, the frequency in blood of NKT cells was much lower than NK cells in our sample, since this subset is usually enriched in tissues.

Altogether, our results indicate a high frequency of activated highly differentiated NK cells in the blood from these patients, with an increase in TIM3 and LAG3 ICs and a low expression of PD-1.

### 3.2. Survival Analysis

The median progression-free survival (PFS) of the cohort, defined as time to progression to immunotherapy, was 10 months (95% CI, 2.81 to 17.19). The median overall survival (OS), defined as time to patient death, was 19 months (95% CI, 11.13 to 26.87).

#### 3.2.1. Patient and Tumor Disease Characteristics

Considering only OS data, tumor stage, reason for treatment indication, type of treatment received and best tumor response achieved reached statistical significance in the univariate analysis. Age showed a trend toward differentiation but did not reach statistical significance. In contrast, there were no differences in OS according to smoking status, sex of patients or tumor histology.

**Figure 1:**
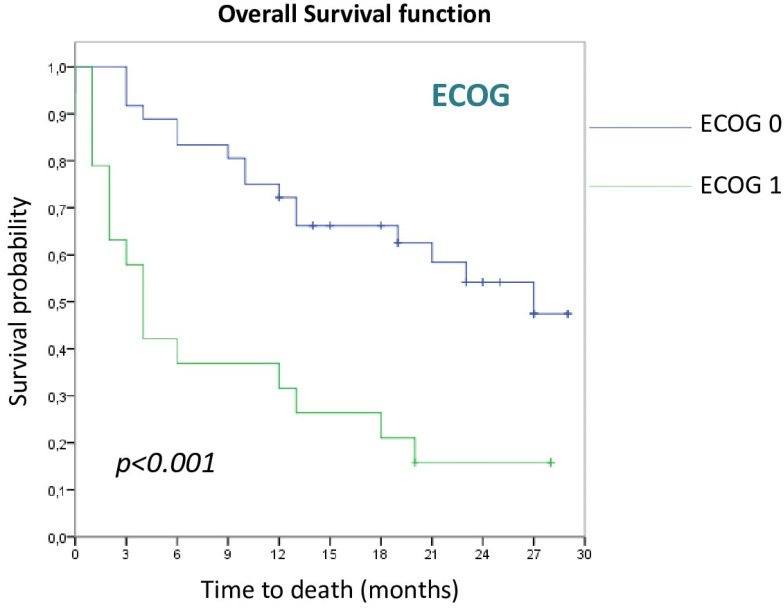
Overall survival curves according to ECOG.

Regarding ECOG scale, those patients with a grade 1 presented worse OS with respect to a grade 0 (4 m vs. 27 m, *p* < 0.001), as can be seen in Figure 1.

**Figure 2:**
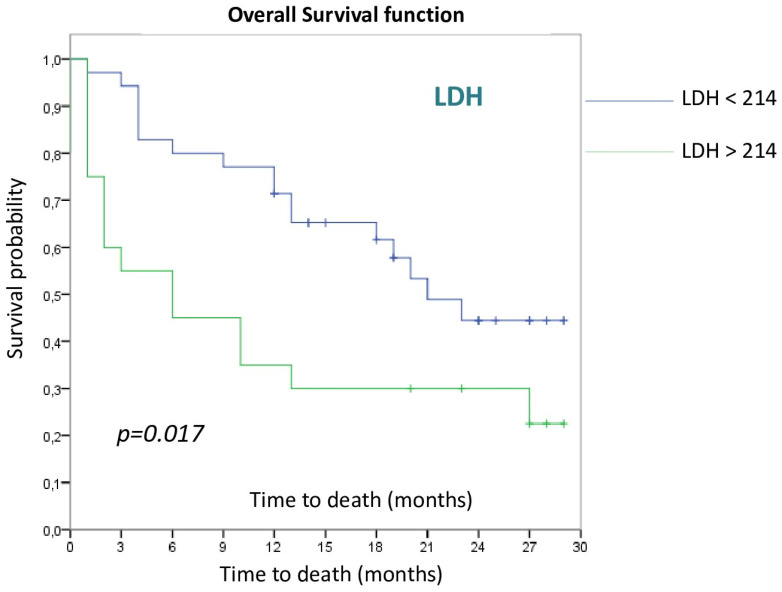
Overall survival curves according to LDH.

Baseline LDH levels also reached statistical significance as a predictor of OS. Those patients with an LDH value above the upper limit of normal (202U/L) had worse OS compared to patients with normal values (6 m vs. 21 m, *p* = 0.017), as is shown in Figure 2.

In contrast, this was not the case for PD-L1 expression level. Here it should be noted that this analysis was performed separating patients in three groups according to PD-L1 expression: negative (less than 1%), intermediate (between 1 and 50%) and high (more than 50%). However, there was a clear trend for increased OS if patients were grouped in negative (less than 1%) vs. positive (more than 1%) PD-L1 irrespectively of the level of positiveness.

#### 3.2.2. Lymphocyte Populations

**Figure 3:**
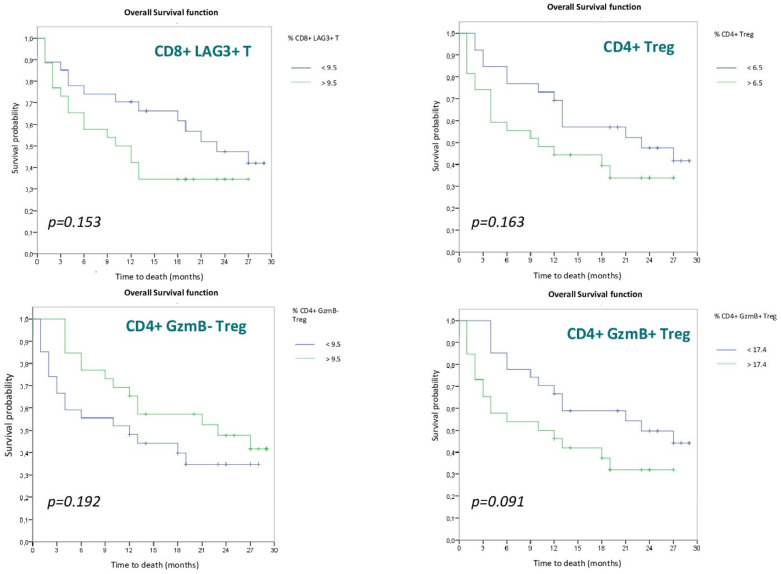
Overall survival curves according to Lymphocyte populations.

To analyze the impact of lymphocyte populations on OS, the cohort was divided into two groups based on the median of the different populations. None of the populations reached statistical significance, although a trend toward differentiation between the two groups was identified in the CD8+ LAG3+ T (*p* = 0.153), CD4+Treg (*p* = 0.163), CD4+GzmB− Treg (*p* = 0.192) and especially in the CD4+GzmB+Treg (*p* = 0.091) cell populations. The Kaplan–Meier OS curves for these four lymphocyte populations are shown in Figure 3.

#### 3.2.3. NK Cell Populations

**Figure 4:**
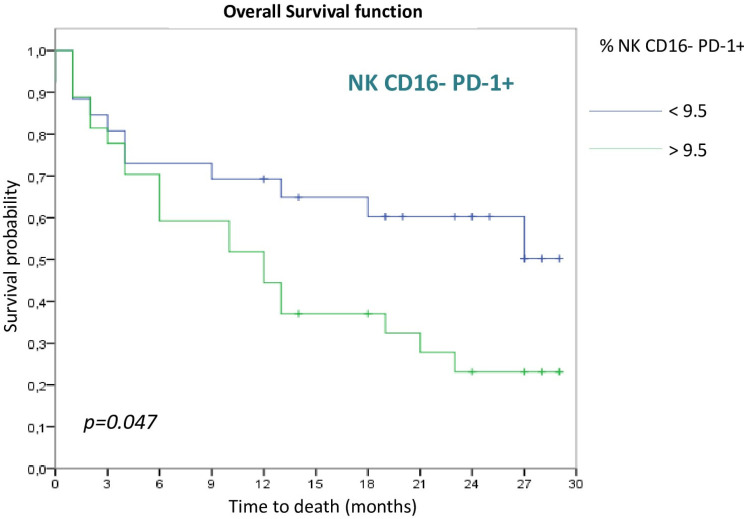
Kaplan Meier curve according to NK CD16− PD-1+.

As for the NK cell populations evaluated, only the NK CD16− PD-1+ population reached statistical significance, with a better OS in the group with a higher frequency of this population (>9.5) (*p* = 0.047) (Figure 4).

There were other NK cell populations that, although they did not reach statistical significance, showed a trend toward differentiation between both groups. These populations are: CD56^dim^ (CD16+) (*p* = 0.082), CD56^dim^ (CD16+) TIM3+ (*p* = 0.153), CD56^dim^ (CD16+) GzmB+ (*p* = 0.15), CD56^dim^ (CD16+) CD57+ (*p* = 0.103), CD56^bright^ (CD16−) (*p* = 0.068) and CD56^bright^ (CD16−) GzmB+ (*p* = 0.085) NK cells.

**Figure 5:**
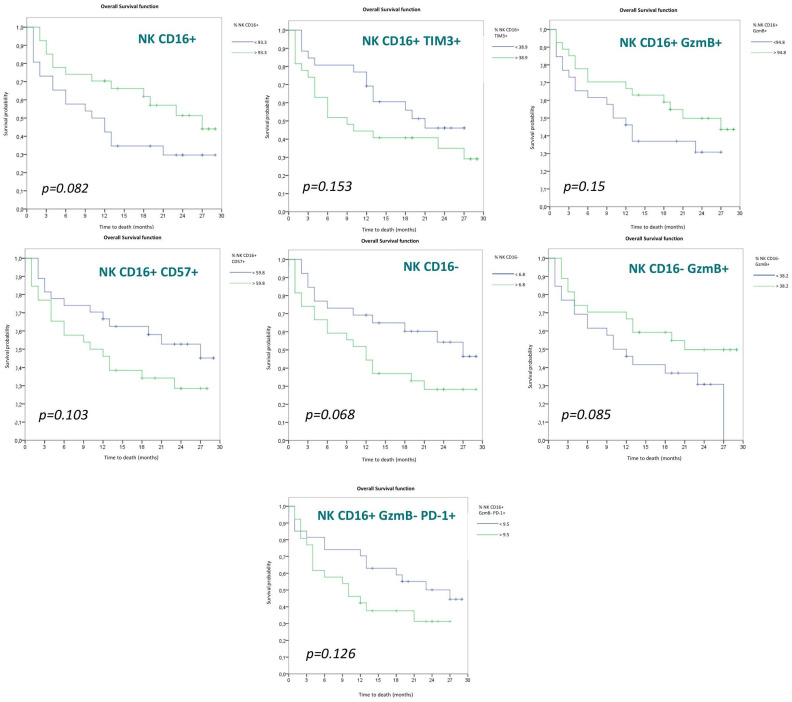
Overall survival curves according to NK populations.

The Kaplan–Meier survival curves for the different NK cell populations are shown below (Figure 5), each divided into two groups according to the mean.

### 3.3. Multivariate Analysis

A Cox regression model was performed to assess the impact of the different variables on OS. The variables selected for inclusion in the model were those that were significant in the univariate survival analysis: ECOG, stage, indication for treatment, type of immunotherapy, best response, LDH and CD56^bright^ PD-1+ NK cells.

The Cox regression results for each of the individual variables are shown below (Table 4).

After performing the multivariate model, the following hazard ratios were obtained (Table 5). As shown in Table 4, the variables that maintained their statistical significance and can predict mortality are ECOG 1 with respect to ECOG 0 (2.5-fold risk) and stage IV with respect to stage III (almost 10-fold risk). In contrast, pembrolizumab treatment (0.25-fold risk) is the only protective factor. The remaining variables not included in this table did not reach significance in the multivariate model and were therefore not considered for this analysis.

## 4. Discussion

Our study prospectively analyzed a cohort of lung cancer patients receiving immunotherapy trying to determine cellular biomarkers in peripheral blood that were predictive of response. A statistically significant difference in OS was found in patients with a lower proportion of NK CD16−PD-1+ cells.

The baseline characteristics of our patients generally coincide with the ones previously described in the literature [24,25,26]. We did observe a high rate of smokers/ex-smokers in our study. This is probably because nonsmoking patients are more likely to have a driver that allows the use of targeted treatment. This makes them noncandidates for immunotherapy, and, therefore, they were not included in our study. This similarity between our data and those described in previous cohorts indicates that there is apparently no bias in the sample selection, supporting its validity to perform in our study.

In terms of observed responses, our sample has an overall response rate (ORR) of 42.4%, a median PFS of 10 months and a median OS of 19 months. All these data are similar to those previously reported in different clinical trials carried out with immunotherapy drugs [27,28,29,30,31,32,33,34,35,36].

Regarding the impact of clinical and demographic variables on OS, the predictive capacity of the ECOG scale stands out, which is congruent with other previously presented studies [37,38]. Likewise, the best response obtained has a very important weight when predicting OS. On the other hand, the type of drug is very likely to be a secondary variable to the stage and line of treatment of the patient. Our results should not be interpreted as a comparative study between drugs.

In terms of LDH expression, statistically significant differences (*p* = 0.017) can be observed depending on the LDH value, with OS being lower in those patients with an LDH value above the upper limit of normality (202U/L). Previous studies have shown that the initial LDH level could be a biomarker related to tumor burden. A higher concentration of this marker is associated with a worse outcome and worse OS in patients treated with ICI [39,40,41].

With respect to circulating T cells, it has been observed in the literature that patients with a good response to immunotherapy tend to have a reduced number of basal cells in peripheral blood, with an expansion of this population at the start of treatment, compared to nonresponders. These results were evaluated in tumors such as melanoma and NSCLC [42,43,44], showing that patients with low levels of CD8+ PD-1+ T cells in peripheral blood at the start of immunotherapy treatment were those with the most durable responses to treatment.

In the case of our study, we tried to better select CD8+ T-cell populations based on PD-1, TIM3 and LAG3 expression. In this regard, despite results in the literature [3,42,43,45], linking high PD-1 expression in CD8+ T cells to better OS and PFS, we did not obtain these results in our sample (*p* = 0.669). The same is true for TIM3 (*p* = 0.730) and LAG3 (*p* = 0.153) expression. However, although the sample size does not allow us to obtain a statistically significant result, we do observe a clear trend toward differentiation with worse OS in those patients with a high proportion of CD8+ LAG3+ T lymphocytes, as already described in the study by Datar et al. [46]. As for GzmB+ expression in CD8+ T cells, although this differentiates them as an activated lymphocyte population, we did not observe any difference in OS in relation to the high or low proportion of these cells in peripheral blood before ICI initiation.

In relation to CD4+ T lymphocytes, there is not as much evidence in the literature, and the latest studies are evaluating the specific contribution of the immunity of this subpopulation to the efficacy of immunotherapy, given that its function is still unknown.

A recently published study of patients with NSCLC on anti-PD-1 therapy found that those who responded to treatment had significantly higher percentages of effector CD4+ T cells (CD62low) prior to treatment initiation (*p* < 0.0001). In contrast, the percentage of Treg lymphocytes (CD25+ FOXP3+ CD4+) was significantly higher in nonresponders (*p* = 0.034) [47]. This study has the largest sample size of those available in the literature (*n* = 143). However, these are patients treated exclusively with nivolumab and do not specify tumor stage. Furthermore, they evaluate the response to treatment with immunotherapy, but not overall survival, so the results may be different from those we have obtained. These data have been confirmed in another cohort of NSCLC patients treated with immunotherapy, showing a higher PFS and higher response in patients with high CD4+ T cells and low Treg at baseline [48]. The sample size of this study is smaller (*n* = 34), and its design is retrospective. On the other hand, it does include any type of immunotherapy, although the cell populations evaluated are less specific than in our study. There are also data from a posthoc analysis of a clinical trial in patients with NSCLC treated with neoadjuvant immunotherapy, which found a relationship between higher baseline CD4+ PD-1+ T-cell levels and a higher response to treatment [49]. A small sample size (29 patients) was included in this posthoc analysis. As a clinical trial, the patients included are likely to be less representative of clinical practice than ours. In addition, the outcome variable assessed is response to immunotherapy, not overall survival. Finally, as a difference with our work, this posthoc analysis included patients with resectable stage III, and immunotherapy was always administered in combination with chemotherapy.

In relation to the data from our study regarding CD4+ T lymphocytes, although there is not enough statistical power to obtain a statistically significant result, we do observe a trend that we will detail below. Regarding CD4+Treg cells, we observed a trend (*p* = 0.163) toward better OS in those patients with a low proportion of these cells in peripheral blood at the start of treatment, with these data being comparable to those obtained in other similar studies and consistent with the anti-inflammatory function of these immune cells [47,48]. Despite the differences between the different studies described above, CD4+Treg cells seem to be associated in all cases with better results, either in terms of response rate or survival time.

GzmB expression by Treg cells is not frequent, and it seems that this expression selects a population of Treg cells that inhibit cell-mediated immunity by cytotoxic mechanisms against CD8+ T cells rather than by secretion of anti-inflammatory cytokines [11]. Analyzing these two cell populations (GzmB+ or not), we also see a trend (*p* = 0.091) to a better OS in those patients with a low proportion of CD4+GzmB+ Treg, supporting a role of this Treg subset in preventing antitumoral cell responses.

In our study, no differences were observed in relation to Tem lymphocytes (both CD8+ and CD4+), confirming previous results found in melanoma patients where different memory T cell subsets increased in TILs from responding patients, but this increase was not observed in blood [23].

Considering the CD8+/CD4+ ratio, it has been shown that a high baseline CD8+/CD4+ ratio in peripheral blood correlates statistically significantly with a better prognosis in early-stage NSCLC (HR 0.19) [50]. However, there are other contradictory data in the literature [51]. A recent study has linked a higher CD4+/CD8+ ratio with a better response and PFS in patients with NSCLC treated with immunotherapy [48].

In the data obtained from our study, the ratio of TCD8+/T CD4+ lymphocytes do not show statistically significant differences, despite previous findings in the literature [38,50,51].

Regarding the NK cell data at the global level, in our study, there were no differences (*p* = 0.512), despite the data from the previously detailed studies [43,52].

If we separate the NK cell population between cytotoxic (CD56^dim^CD16+) and noncytotoxic (CD56 ^bright^CD16−), we observe differences, though without achieving statistical significance. A higher proportion of CD56^dim^NK cells is associated with a higher OS (*p* = 0.082), which somehow agrees with previous studies showing a low proportion of CD56+ and CD16− NK cells (*p* = 0.068) in responder patients [53,54].

Analyzing the expression of different markers within the CD56^dim^NK cell population, we observed that TIM3 expression, without reaching statistical significance, showed a trend (*p* = 0.153) toward greater OS in patients with low TIM3 expression. This could be explained by the fact that TIM3 expression, as with LAG3, confers a distinct functional profile to the NK cell that is associated with a shorter PFS, as demonstrated in the study by Datar et al. [46].

Although not reaching statistical significance, there is also a clear differentiation among CD56+ CD16+ NK patients according to GzmB+ expression. There is a better OS in those patients with a high proportion of CD56+ CD16+GzmB+ NK cells (*p* = 0.15). As previously mentioned, GzmB expression correlates with cell activation, giving the immune cell greater cytotoxic capabilities [14]. In turn, there is a trend toward differentiation in the CD56+ CD16+GzmB− PD-1+ NK population (*p* = 0.126), with a better OS for those patients with a low proportion of this exhausted cell lineage.

As for the other receptors analyzed in the CD56+ CD16+ NK cell population, no differences were observed between the different expression of NKp46, NKG2D and NKG2A (*p* = 0.647, 0.537 and 0.786, respectively), although there are some studies that correlated the concentration of circulating NK NKp46+ CD56^dim^ cells in peripheral blood inversely with prognosis in NSCLC, without analyzing their response to immunotherapy [55].

A trend, although not significant, can be observed in our study regarding CD57 expression, with a better OS for those patients with a low proportion of CD56^dim^CD57+ NK cells (*p* = 0.103). Although recent studies position this cell subtype as a highly differentiated NK cell population with cytotoxic capabilities, CD57 expression can be considered a marker of terminal differentiation with the possibility of anergy and cellular senescence, which would explain the results obtained in our study [56].

Focusing now on the CD56+ CD16− NK cell population, the expression of different markers was analyzed, including LAG3, TIM3, PD-1 and GzmB+. There were no differences in the expression of TIM3, LAG3 and GzmB+, but there is a trend toward differentiation in GzmB+ (*p* = 0.085), with a higher OS in those patients with a high proportion of CD56+ CD16− GzmB+ NK cells. CD56+ CD16− NK cells are regulatory cells and can either activate or inhibit the other immune populations. The CD56+ CD16−GzmB+ NK cell population is difficult to detect in healthy people, but it is increased in people with some pathologies [57], although its exact contribution to the pathology is unclear. The acquisition of cytotoxic capacity of CD56^bright^ NK cells observed in our study could explain that a high proportion of these cells is associated with better OS (*p* = 0.085). Indeed, it has been shown that CD56^bright^ NK cells can acquire cytotoxic properties and antitumoral activity after encountering with specific stimulus [58], and the presence of cytotoxicCD56^bright^ NK cells in TIL correlates with good prognosis in bladder cancer [59].

Interestingly, it was found that the best cell population to differentiate between responders and nonresponders was PD-1+CD56^bright^ NK cells that presented a statistically significant difference (*p* = 0.047). In this case, a lower proportion of PD-1+CD56^bright^ NK cells correlated with a higher OS. One possible explanation for this finding is that a higher PD-1+ expression favors a population that will be highly activated after ICI treatment. Once activated, it might secrete cytokines that might inhibit other cell populations with antitumor cytotoxic capacity.

The main limitation of our study is the small sample size, which probably prevented us from reaching statistical significance for several of the populations studied, which did show a clear trend. The small sample size, together with the variability of some of the populations assessed, results in very large standard deviations. Another limitation is the heterogeneity of the cohort, particularly related to the inclusion of unresectable stage III patients receiving thoracic radiotherapy before starting ICI therapy. Additionally, we have considered only OS results for most of the analysis performed to define predictive biomarkers for immunotherapy.

On the other hand, one of the strengths is the exhaustive characterization of the lymphocyte and NK populations, allowing a high degree of detail in their functional typing.

Although the sample size is small, some clinical variables already known and described in the literature, such as ECOG or tumor stage, reached statistical significance. It is likely that even if it is possible to identify a lymphocyte population with the capacity to predict the OS of patients, it will not have as much weight as other clinical variables. In addition, the difficulty of assessing “response” as a primary endpoint due to the intrinsic characteristics of immunotherapy treatment makes it difficult to assess cell populations as a marker of response.

Studies designed with larger sample sizes should be carried out to see if the promising initial results we have found are confirmed in larger cohorts, helping to optimize the treatment strategy with ICI drugs.

## 5. Conclusions

Lymphocyte and NK cell populations are of great interest as predictive markers for immunotherapy in lung cancer. However, none have yet been identified with sufficient clinical relevance to be established in clinical practice. In our study, PD-1+CD56^bright^ NK cells showed the best predictive ability. It is possible that those cells with high PD1 expression may be activated to a large extent by ICI treatment. Then, more comprehensive trials could demonstrate that our hypothesis improved the unmet need to select better candidates for receiving immunotherapy in clinical practice.

## Figures and Tables

**Table 1:** Population basal characteristics.

Variable	*N* (Total = 55)	%
**Sex**		
Female	16	29.10%
Male	39	70.90%
**Age**		
<75 years	47	85%
>75 years	8	15%
**Smoking habit**		
Never smoker	2	3.60%
Former smoker/Current smoker	53	96.40%
**ECOG**		
ECOG 0	36	65.50%
ECOG 1	19	34.50%
**Histology**		
Squamous	22	40%
Adenocarcinomas	33	60%
**Tumor stage**		
Stage III	16	29.10%
Stage IV	39	70.90%
**Treatment indication**		
Locally advanced	14	25.50%
First line	18	32.70%
Second line or more	23	41.80%
**ICI drug**		
Durvalumab	14	25.50%
Pembrolizumab	21	38.20%
Atezolizumab	18	32.70%
Nivolumab	2	3.60%
**Objective Response**		
Complete response	10	18.80%
Partial response	13	23.60%
Stable disease	12	21.8%
Progressive disease	15	27.30%
Not evaluable	5	9.10%
**LDH**		36.40%
High (>202U/L)	20	36.40%
Normal (<202U/L)	35	63.60%
**PD-L1**		
<1%	10	18.20%
1–49%	21	38.20%
>50%	16	29.10%
Unknow	8	14.50%

ECOG: Eastern Cooperative Oncology Group; ICI: Immune Checkpoint Inhibitor; LDH: Lactate Dehydrogenase.

**Table 2:** Distribution of T lymphocyte populations distribution.

Lymphocyte Population	Median (%)	Standard Deviation (%)
CD8+ T	28.5	25.8
CD8+ PD-1+ T	9.5	18
CD8+ TIM3+ T	4.1	7.2
CD8+ LAG3 + T	9.5	18.4
CD8+ GzmB+ T	31.7	25.5
CD8+ GzmB+ PD-1+ T	1.3	10.7
CD8+ GzmB+ TIM3+ T	1.1	4.6
CD8+ GzmB+ LAG3+ T	2.1	8.8
CD8+ GzmB− T	68.3	25.4
CD8+ GzmB− TIM3+ T	2.2	5.3
CD8+ GzmB− LAG3+ T	6.5	9.6
CD8+ GzmB− PD-1+ T	4.7	6.7
CD8+ Tem	3.5	9.5
CD4+ T	29.6	24.4
CD4+ Treg	6.5	8.8
CD4+ GzmB− Treg	82.6	24.4
CD4+ GzmB+Treg	17.4	24.4
Coeficient CD8+ T/CD4+ T	1	2.3

**Table 3:** Distribution of NK cell subsets.

NK Cell Populations	Median (%)	Standard Deviation (%)
NK (CD56+CD3−)	15.7	11.4
NKT (CD56+CD3+)	7.5	9.2
NK CD16+	93.3	17.5
NK CD16+ TIM3+	38.9	24.3
NK CD16+ LAG3+	16.6	25
NK CD16+ PD-1+	5.9	15.3
NK CD16+ GzmB+	94.8	18.6
NK CD16+ GzmB+ TIM3+	32.3	20.7
NK CD16+ GzmB+ LAG3+	21.3	19.1
NK CD16+ GzmB+ PD-1+	5.3	17.8
NK CD16+ GzmB−	4.1	17.4
NK CD16+ GzmB− TIM3+	1	8.1
NK CD16+ GzmB− LAG3+	0.1	8.6
NK CD16+ GzmB− PD-1+	0.2	5.4
NK CD16+ CD57+	59.8	17.9
NK CD16+ NKp46+	74.2	23.4
NK CD16+ NKG2D+	69	33
NK CD16+ NKG2A+	45.9	21.9
NK CD16−	6.8	17.5
NK CD16− GzmB+	38.2	39.7
NK CD16− PD-1+	9.5	15.3
NK CD16− LAG3+	5	13.8
NK CD16− TIM3+	40.7	22.5

**Table 4:** Individual coefficients of the variables included in the Cox regression.

Covariates	HR	CI (HR) 95%	*p*-Value
ECOG 1	3.247	1.606–6.562	0.001
Stage IV	6.539	1.983–21.565	0.002
TI locally advanced	-	-	0.001
TI first line	5.063	1.108–23.126	0.036
TI second line and more	12.840	2.962–55.656	0.001
Durvalumab	-	-	0.001
Nivolumab	3.336	0.302–36.831	0.325
Pembrolizumab	5.668	1.266–25.376	0.023
Atezolizumab	15.635	3.543–68.922	<0.001
Complete response	-	-	<0.001
Partial response	4.582	0.534–39.348	0.165
Stable disease	8.384	1.026–68.509	0.047
Progressive disease	37.880	4.830–297.063	0.001
Not evaluable	377.216	33.191–4287.125	<0.001
High LDH > 214 U/L	2.262	1.124–4.551	0.022
NK CD16- PD-1+ (>9.5)	2.052	0.980–4.295	0.056

ECOG: Eastern Cooperative Oncology Group; TI: Treatment indication; LDH: Lactate dehydrogenase.

**Table 5:** Multivariate analysis.

Covariates	HR	HR 95% CI	*p*-Value
ECOG 1	2.496	1.155–5.391	0.02
Stage IV	9.929	1.193–82.625	0.034
Durvalumab	0.454	0.037–5.540	0.536
Nivolumab	0.199	0.024–1.657	0.135
Pembrolizumab	0.233	0.098–0.557	0.001
Atezolizumab	-	-	0.008

ECOG: Eastern Cooperative Oncology Group; HR: Hazard ratio; CI: Confidence interval.

## Data Availability

The data presented in this study are available on request from the corresponding author.

## Supplementary Materials

The following supporting information can be downloaded at: https://www.mdpi.com/article/10.3390/cancers15020329/s1, Table S1: list of antibodies used for the phenotyping of immune cells. Figure S1: Flow cytometry gating strategy.
