# Supplementary material for: A Subset of PD-1-Expressing CD56bright NK Cells Identifies Patients with Good Response to Immune Checkpoint Inhibitors in Lung Cancer
Source: Cancers (Basel). 2023 Jan 4;15(2):329. doi: 10.3390/cancers15020329 (PMC9856517; doi:10.3390/cancers15020329)
Supplement: Supplementary file 1 [file cancers-15-00329-s001.zip › Supplementary table S1.pdf]

Supplementary table S1. List of antibodies used for the phenotyping of immune cells

Antibody	Fluorophore	Comercial brand	Reference
CD16	FITC	Miltenyi	130-113-392
GzmB	PE	Miltenyi	130-116-486
CD56	PerCP Vio700	Miltenyi	130-114-551
NKG2A	PE-Vio770	Miltenyi	130-113-567
NKp46	APC	Miltenyi	130-092-609
CD57	APC-Vio 770	Miltenyi	130-11-813
NKG2D	BV421	BD	743558
CD3	VioGreen	Miltenyi	130-113-134
TIM3	PE-Vio770	Biolegend	345014
LAG3	APC	Miltenyi	130-105-453
PD1	Alexa Fluor700	Biolegend	329952
CD8	FITC	Miltenyi	170-078-007
FoxP3	FITC	Biolegend	320106
CD25	APC	Miltenyi	130-113-280
CD4	VioBlue	Miltenyi	130-113-258
CD27	PE	Miltenyi	130-113-630
CD45RO	PE-Vio770	Miltenyi	130-113-551
CCR7	APC	Miltenyi	130-108-286
