# Supplementary figures and images for: A Subset of PD-1-Expressing CD56bright NK Cells Identifies Patients with Good Response to Immune Checkpoint Inhibitors in Lung Cancer
Source: Cancers (Basel). 2023 Jan 4;15(2):329. doi: 10.3390/cancers15020329 (PMC9856517; doi:10.3390/cancers15020329)

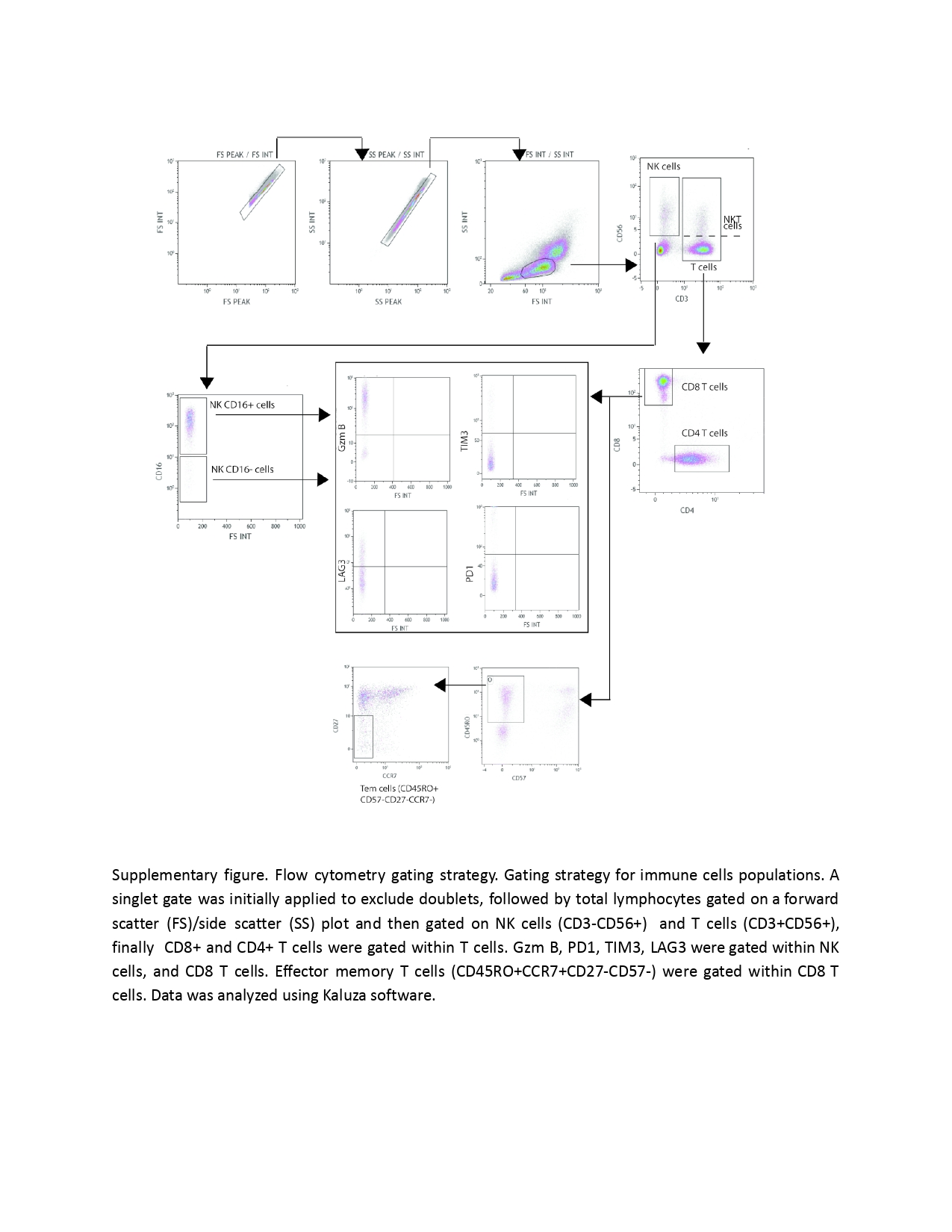

Supplement: Supplementary file 1 [file cancers-15-00329-s001.zip › Supplementary Figure S1.jpg]
